# Methotrexate-associated Hodgkin Lymphoma Occurring Decades after Methotrexate Initiation

**DOI:** 10.31662/jmaj.2025-0117

**Published:** 2025-06-20

**Authors:** Takahiro Kobayashi, Yosuke Ono, Nobutaka Hirooka

**Affiliations:** 1 Department of General Medicine, National Defense Medical College, Saitama, Japan; 2 Yokota Airbase Hospital, 374^th^ Medical Group, United States Airforce, Tokyo, Japan

**Keywords:** methotrexate-associated lymphoproliferative disorder, Hodgkin lymphoma, rheumatoid arthritis

An 84-year-old man with a 40-year history of rheumatoid arthritis (RA), treated with methotrexate (MTX) for decades, presented with an eight-month history of intermittent fever and fatigue. Physical examination revealed lymphadenopathy and RA-associated finger deformities. Laboratory tests showed elevated levels of soluble interleukin-2 receptor (8,452.7 U/mL, reference range: 157-474) and C-reactive protein (11.7 mg/dL), as well as a decreased lymphocyte count. Chest computed tomography revealed multiple enlarged lymph nodes in the axillary, peritoneal, para-aortic, and mediastinal regions (Figure 1). MTX was immediately discontinued, and a subsequent pathological investigation of an inguinal lymph node showed large malignant lymphocytes with positive Epstein-Barr virus (EBV)-encoded small ribonucleic acid and Reed-Sternberg cells, indicating a diagnosis of stage Ⅲ mixed cellularity classic Hodgkin lymphoma. The patient’s EBV-related antibody status was: anti-viral capsid antigen (VCA) immunoglobulin (Ig)M (-), VCA IgG (+), EBV nuclear antigen IgG (+), suggesting a past EBV infection. Discontinuation of MTX resolved the fever within 1 week and was followed by a reduction in lymph node size over 5 months (Figure 2). This lymphoma is classified as MTX-associated lymphoproliferative disorder (MTX-LPD). According to the revised 4th *WHO Classification of Tumors of Hematopoietic and Lymphoid Tissues*, MTX-LPD falls under the category of other iatrogenic immunodeficiency-associated lymphoproliferative disorders ^(1)^. Hodgkin lymphoma is a common pathological finding seen in MTX-LPD, following diffuse large B-cell lymphoma ^(2)^. MTX-LPD can be managed without chemotherapy, as remission can be achieved in 50-60% of the cases by simply eliminating MTX ^(2), (3)^. As seen in this case, EBV plays a substantial role in the pathogenesis of MTX-LPD, even in the latent phase following a prior infection ^(4)^. There is growing awareness of MTX-LPD because of its Asian-dominant prevalence ^(2)^, and MTX-LPD should be considered regardless of the size and distribution of lymphadenopathy, or the duration of MTX administration, even for decades, as in this case. Furthermore, the prompt discontinuation of MTX prior to pathologic diagnosis, as was implemented in this case, is crucial for rapid diagnosis without overlooking self-limiting conditions.

**Figure 1.**
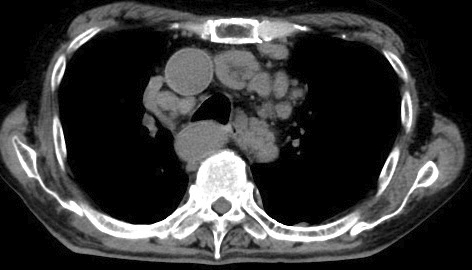
Multiple swollen lymph nodes at the mediastinum.

**Figure 2.**
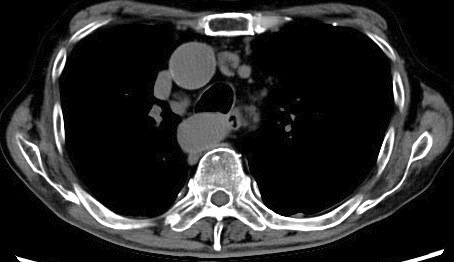
Shrunken lymph nodes 5 months later.

## Article Information

### Conflicts of Interest

None

### Author Contributions

Takahiro Kobayashi wrote the first draft of the manuscript. Yosuke Ono and Nobutaka Hirooka revised the manuscript. Takahiro Kobayashi, Yosuke Ono, and Nobutaka Hirooka contributed to the patient care. Takahiro Kobayashi organized the manuscript.

### Approval by Institutional Review Board (IRB)

Not applicable.

### Informed Consent

The written consent form was obtained from the patient about publishing manuscripts and photographs.
